# Exploring the attitudes of health science students in Spain and Bolivia towards death. A cross sectional survey

**DOI:** 10.1186/s12904-020-00615-z

**Published:** 2020-07-21

**Authors:** Sagrario Pérez- de la Cruz, Ivonne Ramírez

**Affiliations:** 1 Department of Nursing, Physiotherapy and Medicine, University of Almería, Crta del Sacramento s/n, La Cañada de San Urbano, 04250 Almería, Spain; 2 University of San Francisco Xavier de Chuquisaca, Sucre, Bolivia

**Keywords:** Students, End-of-life, Education, Medicine, Nursing, Physiotherapy

## Abstract

**Background:**

One of the most difficult and stressful tasks faced by health science students is having to cope with death and dying due to the emotional burden of the same. Furthermore, the moral, ethical and professional values of future health professionals are influenced by the cultures where they live.

**Purpose:**

This study sought to compare and analyze the perception on end of life among a sample of health science students in Spain and Bolivia.

**Methods:**

A descriptive, cross-sectional and multi-centric study. The total sample (548 students) was comprised of three groups: medical, nursing and physiotherapy students, of whom 245 were from Bolivia, and 303 were Spanish students. The measurement instruments used were the Bugen’s Coping with Death Scale and the Death Self-Efficacy Scale by Robbins.

**Results:**

No statistically significant differences were observed between Spanish and Bolivian students (*t* (546) = − 0.248, *p* = 0.804) using the Bugen scale. This implies that there are no differences between the perception of both groups of students and that both groups use similar strategies to cope with death. Additionally, the beliefs and attitudes of both groups were similar, with Bolivian students presenting a trend towards improved scores. No differences were found between Spain and Bolivia in the results obtained on the Robbins scale, with students from both countries displaying similar skills and capabilities for facing death.

**Conclusions:**

The beliefs on death of health science students from Spain and Bolivia were not affected by the respective cultures, type of degree studied, students’ age, or the country of origin, however, we found that students in Bolivia value death as something more natural than their Spanish counterparts.

**Practice implications:**

To appropriately prepare students for this topic, education on coping with death and dying must be included within the university curriculum.

**Trial registration:**

2016DEC018.

## Background

Coping with death and the end of life-death process is one of the most difficult and stressful tasks faced by health science students [1, 2]. Upon qualification, students must be prepared to face situations related with palliative care and end of life and know how to appropriately respond to these circumstances. Furthermore, professionals must deal with the burden of communicating bad news to the family and the patient throughout the end-of-life process. In particular, nursing professionals are traditionally more actively involved in the dying process whereas, for physicians or medical practitioners, the subject of death is generally experienced from a different perspective [2, 3]. Theoretically, physicians professionals are best prepared for the management of patient death [3, 4]. However, this is not always the case, as frequently reflected in the limited direct care provided to patients by their physicians. Physiotherapists and allied health professionals also deal with these patients, accompanying them on many occasions until the end of their lives by providing treatment and care [5].

Thus, in order to provide the best possible care to terminal patients and those with advanced illnesses (and their family members), specific training is necessary regarding the processes of mourning, death, terminal disease, and palliative care, both within the university curriculum, as well as during professional development [2]. In particular, this should be reflected in the academic curriculum of student nurses and doctors [2, 4]. However, in Bolivia this type of content is not taught, and in Spain there is no consensus on the hours of training nor contents related to end of life and how future professionals should face the same. Furthermore, in Spain this specific training is an optional course subject. A scarce number of studies have been published on this topic, and fewer still are available featuring health science students of different degrees from an international perspective.

In addition, it is important to consider that the cultural context, globalization and the progress in technology and medicine, all influence the ethical values that underlie healthcare and the performance of health professionals, facilitating their daily work and expanding the life expectancy of the patients [6, 7]. A study by Alfred et al. [8] discovered that the moral, ethical and professional values of nursing professionals are influenced by the cultures where they live, learn and practice. The values or ideals that both professionals and students experience if they study abroad influences their subsequent integration into the workplace. Other authors, such as Soto- Pérez-de-Celis et al. [6] declared that globalization may accelerate cultural development, proposing the development of a universal ethical code for the nursing profession, across various cultures. These authors found that cultural values play a fundamental and substantial role in the moral actions of health professionals.

This line of research has been the object of several studies [7–9]. Rassin [9] compared the values between Israeli and Russian students while Pang [7] compared the values between Chinese, American and Japanese nurses, and between nurses in Taiwan and the USA. These studies found that training and previous values of these students influenced the way that they faced death, finding that the values were different among these countries and this may have modified the way end of life is faced. This study is also unique as it compared students from three degrees between two geographically distant countries. Few studies have been conducted in Latin America addressing this gap, although there are similarities among Latin American countries, as we have studies analyzing small groups of nursing or psychology students in how they cope with death, however, these studies have not been extrapolated to international publications [4, 6, 9]. This study adds to the evidence base on the importance of comparisons between geographically distant countries to identify the possible influence of culture. Thus, research has shown that palliative care must be culturally sensitive in its delivery and this is influenced by the way it is delivered/educated.

This study features health science students in Spain and Bolivia, two countries with similar language and concepts (religion, common history), although geographically very distant. One can assume that considering the different customs of each country, students from both countries may be different in the way they face death and dying. This was our study hypothesis.

### Purpose

The aim of this study was to compare and analyze the attitudes on death among a sample of health sciences students in Spain and Bolivia.

## Methods

This study was based on a descriptive, cross-sectional, observational and multicenter design.

### Sample

The study population comprised students of three different health science degrees: medicine, nursing and physiotherapy. Recruitment took place from November 2017 and June 2018.

The inclusion criteria for this study were: 1) mid-degree university students in their second and third year of health science studies, 2) students who voluntarily agreed to participate in the study and who signed the informed consent, and 3) students who regularly attended classes. Students who held jobs alongside performing their university studies were excluded, together with those who were in the first and final year of their studies and those that failed to complete the questionnaires in the assigned time period. We selected students in intermediate years of their degree in order have a more ‘neutral’ sample, meaning that, during the first years of study, students still have not had any contact with patients in these stages, whereas students in their last year most likely have already been trained to face these situations in a certain manner, which would introduce a bias effect on the data. Physiotherapy students were also included, as these are students who also study at the health sciences faculties of both countries, which facilitated access to this population; likewise, these are future professionals who will also experience the end of life of their patients with degenerative illnesses.

**Fig. 1:**
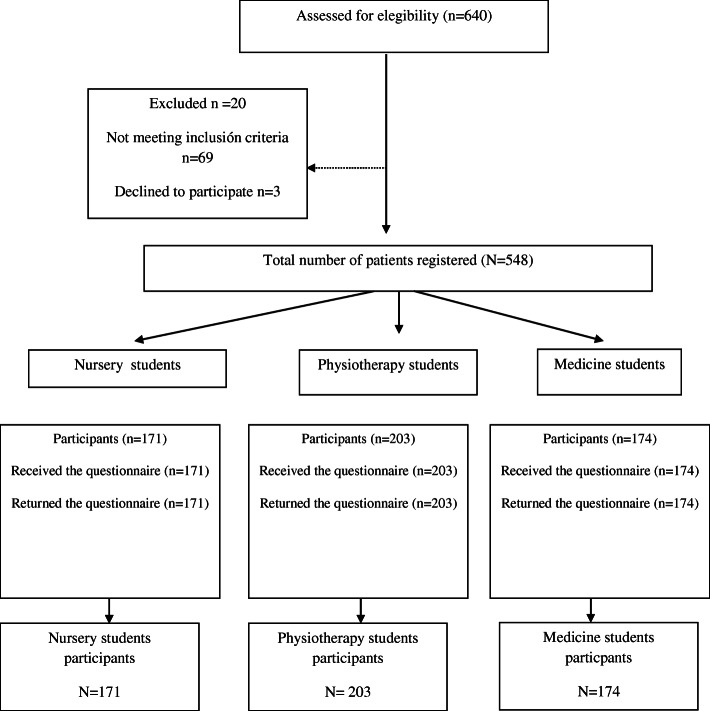
Study design flowchart

The neutral sample selection process is shown below in Fig. 1.

### Data collection

The data collection period was from January to June 2018. As this was a geographically dispersed sample (with different faculties and two geographically separated countries), contact was made directly with those teachers responsible for subjects that were being taught during the teaching period. One of the members of the research team (SPC) was in charge of contacting the Spanish universities, whereas another researcher (IFR) contacted the universities in Bolivia. Once a response was received, a day, time and place was arranged to be able to present the participants with the questionnaires in person. These questionnaires were issued during those classes which had the largest attendance, and the students had several days’ notice in order to take part in the study which, in turn, ensured the greatest sample number possible. In both Spain and Bolivia, one person (MRD in Spain and IJL in Bolivia) was in charge of delivering and collecting the questionnaires, both of whom were external to the research (in order to avoid influencing the data and to maintain impartiality).

Considering that the questionnaire given to participants required at least 15 min time to complete the scales, the teachers of the subjects dedicated half their class period (approximately 30 min) towards explaining to the participating students what the study was about and answering any questions that arose during the completion of the same. The self-administrated questionnaires were anonymous. The students were granted sufficient time (30 min) to answer all questions with ease.

### Instruments used for data collection

The Bugen Coping with Death Scale [10] aims to evaluate the competency for dealing with death. This scale includes items which examine the presence of a series of skills required for facing death, as well as the beliefs and attitudes regarding the same. These include dealing with the emotions anticipated during processes of mourning, planning a funeral, preparing for facing death of oneself and that of loved ones, skills for helping people who are in the process of mourning and how to reach closure. Higher scores suggest better coping strategies for death. Scoring is based on a Likert scale ranging from 1 (totally in disagreement) to 7 (totally in agreement). The total score for coping with death is obtained via adding the scores obtained on each item [10, 11].

The second scale used was the Death Self-Efficacy Scale by Robbins. This focuses on the perceived control that a subject has regarding the process of dying, based on their own knowledge or on their environment. This tool consists of 44 items, scored from between 0 to 100 in units of 10, with 0 being very insecure and 100 feeling very confident of their response. For the present study, the scale was modified, using 11 items, as the remainder did not comply with the aims of the research. Regarding the psychometric properties of the scale, the original version has reportedly had an internal consistency of 0.95, *p* <  0.001. In addition, this scale has shown a good test-retest stability of 0.91, p <  0.001. This scale evaluates the overall control that a subject suspects he, or she, may have over this or over the person’s own behaviors, plus the control that the subject believes their behavior has upon the surrounding stimuli [11, 12].

These scales were selected for this study based on previous studies related with end of life and health sciences professionals. Furthermore, these are validated scales, with validated translations into Spanish, therefore measurement bias is largely eliminated, in order to accurately reflect the reality that this study seeks to assess [11].

### Statistical analysis

The data were tabulated using IBM® SPSS® Statistics V21.0.0 software. The socio-demographic variables of participants were analyzed, including their sex, age, studies, and place of residence. Frequencies, percentages, means and typical deviations were described. The Mann-Whitney test was used to examine the differences between groups.

The non-parametric tests employed were the Wilcoxon-Mann-Whitney test (for two independent samples), the Kruskal-Wallis test (for k-independent samples) and the χ-squared test (for two qualitative independent samples). In all cases, the results were calculated based on a 95% confidence interval.

## Results

The final sample comprised 548 students of both sexes, of which 245 were in their second, third and fourth year of studying medicine, nursing and physiotherapy at two Bolivian universities. The second group comprised 303 students of medicine, physiotherapy and nursing who were in their second to third year and attended three Spanish universities. In total, 365 students were female (66.60%) and 183 students were male (33.39%). The mean age of the sample was 20.7 years. The demographic characteristics of the sample are featured below (Table 1).

**Table 1:** Socio- demographic characteristics of the study sample

	Total (*n* = 548)	Country	
		Spain (*n* = 303)	Bolivia (*n* = 245)
Age, *mean (SD)*	20.7 (2.7)	20.9 (3.1)	20.5 (1.9)
Gender, *n (%)*			
Female	365 (66.6)	197 (65)	168 (68.6)
Male	183 (33.4)	106 (35)	77 (31.4)
Population of place of residence, *n (%)*			
< 5000	91 (16.6)	44 (14.5)	47 (19.2)
5000–25,000	160 (29.2)	92 (30.4)	68 (27.8)
25,000–100,000	119 (21.7)	80 (26.4)	39 (15.9)
> 100,000	178 (32.5)	87 (28.7)	91 (37.1)
Degree, *n (%)*			
Nursing	171 (31.2)	96 (31.7)	75 (30.6)
Physiotherapy	203 (37)	130 (42.9)	73 (29.8)
Medicine	174 (31.8)	77 (25.4)	97 (39.6)
Response rate, n (%)			
Nursing		96 (99)	75 (100)
Physiotherapy		130 (100)	73 (98)
Medicine		77 (99)	97 (99)

Table 2 displays the description and comparison of the scores for the items on the Bugen scale for both Spanish and Bolivian students. For the items: ‘I feel prepared to face my dying process’ (EB1) (*t* (546) = − 5.728, *p* <  0.001); ‘I feel prepared to face my death’ (EB2) (*t* (546) = − 4.472, *p* <  0.001); ‘I can express my fears about dying’ (EB3) (*t* (546) = 3.946, *p* <  0.001); and ‘I can help someone with their thoughts and feelings about death and dying’ (EB10) (*t* (546) = − 4.369, *p* <  0.001), statistically significant differences were observed, whereas for the remaining items on this scale there were no differences noted.

**Table 2:** Description and comparison of items on the Bugen scale by country

	Country		Difference means	Student’s t-test	
	Spain (n = 303)	Bolivia (n = 245)		*t*(546)	*p*-value
**EB1** I feel prepared to face my dying process	3.31 (1.89)	4.25 (1.94)	−0.94	−5.728	< 0.001
**EB2** I feel prepared to face my death	3.18 (1.89)	3.94 (2.07)	−0.76	−4.472	< 0.001
**EB3** I can express my fears about dying	4.73 (1.65)	4.15 (1.82)	0.58	3.946	< 0.001
**EB4** I can talk about my death with family and friends	459 (1.88)	4.03 (2.17)	0.56	3.246	0.001
**EB5** I will be able to cope with future losses	3.63 (1.85)	3.76 (2.13)	−0.13	−0.762	0.446
**EB6** I feel able to handle the death of others close to me	3.33 (1.81)	3.4 (2.05)	−0.07	−0.429	0.668
**EB7** I know how to listen to others	5.11 (1.41)	4.74 (1.94)	0.37	2.531	0.012
**EB8** I know how to speak to children about death	3.42 (1.71)	3.84 (2.03)	−0.42	−2.666	0.008
**EB9** I am able to spend time with the dying if I need to	4.33 (1.78)	3.98 (1.99)	0.35	2.15	0.032
**EB10** I can help someone with their thoughts and feelings about death and dying	3.69 (1.68)	4.35 (1.85)	−0.66	−4.369	< 0.001
**EB11** I would be able to talk to a friend or family member about their death	5.2 (1.72)	4.68 (1.9)	0.52	3.371	0.001
**EB12** I can communicate with the dying	4.77 (1.58)	4.44 (1.93)	0.33	2.141	0.033

**Fig. 2:**
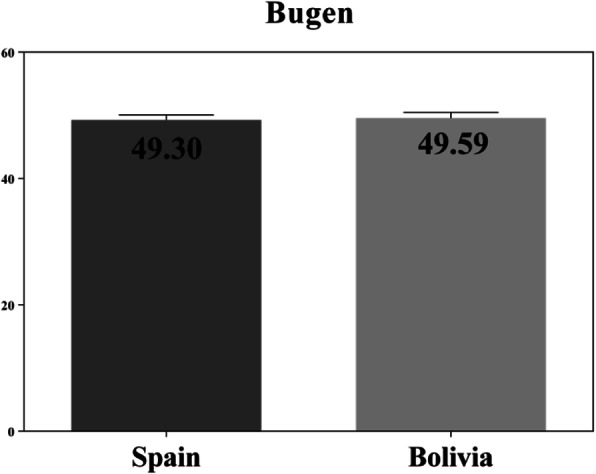
Total values (percentage) Bugen scale

For the total score of the scale (Fig. 2), no statistically significant differences were observed between the Spanish and Bolivian students (*t* (546) = − 0.248, *p* = 0.804), implying that there are no differences between the perception of students in Spain and Bolivia. Furthermore, both groups used similar strategies, beliefs and attitudes to face death, with Bolivian students presenting a non-significant trend towards improved scores compared to Spanish students.

Table 3 displays the descriptive analysis and comparison of the scores of the items on the Robbins scale for both Spanish and Bolivian students. For the items: ‘facing the death of an older person’ (EH1); ‘listening to the concerns of a dying patient’ (EH2); ‘listening to the concerns of a family member of a dying patient’ (EH3); ‘communicating with a dying patient’ (EH6); ‘coping with the death of a friend your age and accompanying a person at the time of their death’ (EH8 and EH9), statistically significant differences were observed among students from both countries, whereas for the remaining dimensions no differences were found.

**Table 3:** Descriptive analysis and comparison of items on the Robbins scale, by country

	Country		Difference means	Student’s t-test	
	Spain (n = 303)	Bolivia (n = 245)		*t*(546)	*p*-value
**EH1** Coping with the death of an older person	7 (2.29)	6.02 (2.99)	0.98	4.326	< 0.001
**EH2** Listening to the concerns of a dying patient	7.18 (1.99)	6.31 (3.07)	0.87	4.019	< 0.001
**EH3** Listening to the concerns of a family member of a dying patient	7.44 (2.06)	6.46 (2.95)	0.98	4.557	< 0.001
**EH4** Touching a cadaver	6.98 (3.04)	6.93 (3.28)	0.05	0.199	0.842
**EH5** Providing physical care to a dying patient	7.4 (2.12)	6.8 (2.85)	0.6	2.797	0.005
**EH6** Communicating with a dying patient	7.41 (2.02)	6.52 (2.93)	0.89	4.183	< 0.001
**EH7** Expressing condolences to a patient’s family	7.25 (2.49)	7.01 (2.99)	0.24	1.02	0.308
**EH8** Coping with the death of a friend your age	3.82 (2.71)	6.05 (3.24)	−2.23	−8.782	< 0.001
**EH9** Accompanying a person at the time of their death	5.97 (2.73)	6.99 (2.99)	−1.02	−4.177	< 0.001
**EH10** Being with a person who presents unpleasant physical symptoms	6.83 (2.23)	6.52 (2.92)	0.31	1.423	0.155
**EH11** Providing emotional support to a patient’s family	7.31 (2.02)	7.42 (2.78)	−0.11	−0.573	0.567

**Fig. 3:**
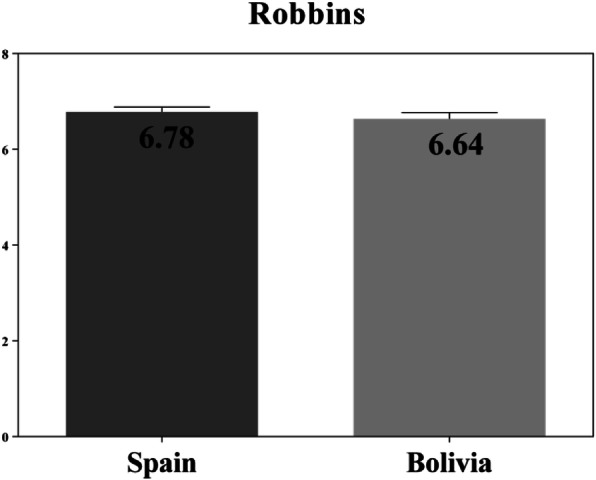
Total values (percentage) Robbins scale

On the total score of the scale (Fig. 3) no statistically significant differences were observed between Spanish and Bolivian students (*t* (546) = − 0.248, *p* = 0.804). In the case of Spanish students, slightly improved scores were obtained, although these were not statistically significant.

## Discussion

The studies available to date focusing on the process of dying and death reflect the cultural, social and religious difficulties faced by health professionals caring for and accompanying patients and family members during this process [2, 13, 14].

Death can be considered a taboo subject in some cultures, however research suggests that healthcare professionals and students want to participate in the accompaniment of end-of-life processes, however, they are unsure of how to do so [15, 16].

Another very important aspect during this transition period (life-death), highlighted by Benbunan-Bentata et al. [17], is the effects of the emotions displayed by health professionals (in our case, future health professionals) in relation to possible anxiety and stress derived from this situation. Thus, it is necessary to practice communication and information skills, provide psychosocial support, include assessments of needs and assign importance to bioethical aspects and interdisciplinary work among professionals, and, more specifically. Among medical and nursing professions. Students and professionals should not only be trained in technical-scientific knowledge. Rather, they must respond appropriately, demonstrating a sensitivity to human suffering. Our findings confirm the importance of dealing with this issue among health science students of Spain and Bolivia and the need to develop emotional skills towards death and the process of dying among these students during their degree studies, without necessarily having to wait until professional practice.

In the Latin American context, Mercado and García [18], and Dueñas, Canchote and Tovilla [19] researched the attitude towards death and its relationship with medical empathy among medical students and found that the attitude towards death of these students was statistically significant [18]. Also, the previously mentioned study by Mercado and García found that 82.7% of students believed that they would have to face death more frequently when practicing their profession, whereas 78.7% defined death as something natural and 40.4% showed a tendency towards denial, even though they had had a close experience to death [19]. There is a clear agreement regarding the academic education that students feel they should have on this topic during their university degree studies, according to the first two first items included on the Bugen scale, exploring the need to be trained in facing end of life (90% of the sample). Cardozo and Sosa [20] conducted a study with students in their final year of medicine to attempt to identify how students cope with the situation of death/dying and what level of training they received during their studies. Up to 82.7% of those surveyed believed that they would have to frequently face death while practicing their profession, although the percentage that defines death as something natural decreased to 78.7%. These results are related to our findings, confirming the need to approach this issue with health science students of Spain and Bolivia. This demonstrates the importance of developing emotional skills towards death and the process of dying among health sciences students during their degree studies, without necessarily having to wait until professional practice.

Other studies, within the field of psychology, have demonstrated the presence of stress among students regarding the topic of death in their profession. These studies concluded that there was a need to work on the conception of dignity at the time of death, meaning both that a doctor-patient collaboration is necessary and that the importance of the humanistic field in the education of future professionals should be highlighted [21, 22]. A further study [23] determined that the anxiety displayed by these students towards death was also closely related to their personal and cultural background. The results obtained in the present study regarding the Robbins scale indicate a lack of significant differences among the students of Bolivia and Spain, regarding how to appropriately manage the end of life processes of their patients, although it is necessary to improve how students respond to these situations.

In the case of nursing students, Espinoza and Sanhueza [24] sought to explore the fear of death and its relationship with emotional intelligence among students in their final years of nursing studies. These authors concluded that high levels of emotional intelligence were associated with less fear of death. This demonstrates the need to develop emotional skills towards death and the process of dying among health sciences students during their degree studies, without necessarily having to wait until professional practice.

In Latin America, various authors have researched attitudes towards death, and the related anxiety, among professionals throughout the health sector, including those who work in palliative care [25, 26]. Thus, appropriate information and experiential exercises can modify a person’s attitude towards death, desensitizing and/or de-dramatizing associated fears. According to the previously mentioned study, it is important to reflect on death and dying rather than avoiding the subject, or only considering it when related to advanced age and the evolution of the person [26].

This analysis aimed to analyze and verify the hypothesis that the attitudes of health professionals in Latin America in situations of death and suffering can influence the quality of the care received by patients [18, 20, 26]. The first experiences related with death during clinical placements have been found to constitute one of the most stressful events for nursing students and demonstrate how, from a teaching perspective, it is necessary to teach future professionals coping strategies to enable them to effectively manage these situations [25].

### Study limitations

One of the main limitations of the present study is related with the risk of bias in the selection of the sample, however there is an intention to minimize this fact via the selection of students within the same age range and with similar socio-demographic characteristics. Likewise, it would be interesting to perform the same study with health science students who may have already had more continuous contact with patients, in order to allow these to express their opinion more appropriately, considering the lived experience. The results of this study do not necessarily reflect other international contexts, and therefore, it would be interesting to compare these findings with similar cultural and social realities, in order to further support these findings.

## Conclusion

No significant differences were observed between Spanish and Bolivian students in their attitudes towards death. The academic curriculum of health professionals should assign greater importance to these issues during university education so that students are better prepared to face cases of patient death with both professionalism and humanism.

Future studies, with larger samples, should research this topic in further depth to identify the educational needs of health science students on end of life and the death of patients. Furthermore, it is necessary to analyze in depth whether there are differences in the educational programs of both countries. In addition, it would be interesting to consider other features that may be different between both groups, despite the fact that they share a similar language and culture.

## Data Availability

Datasets from this study are not available since we do not have the consent to share the data neither from the Ethical Research Board nor from the participants.

## Abbreviations

SPSS: Statistical Package for the Social Sciences
SD: Standard Deviation

## References

[1] Corr CA. Death in modern society. En Doyle D, Hanks G W C y , MacDonald N (Eds). Oxford texbook of palliative medicine. Oxford: Oxford Medical Publications (pp. 28–36); 1983.

[2] Tomás Sábado J, GuixLlistuella E (2001). Ansiedad ante la muerte: Efectos de un curso de formación en enfermeras y auxiliaries de enfermería. Enfermería Clínica.

[3] Redinbaugh EM, Sullivan AM, Block SD, Gadmer NM, Lakoma M, Mitchell AM, Seltzer D, Wolford J, Arnold RM (2003). Doctors’ emotional reactions to recent death of a patient: cross sectional study of hospital doctors. BMJ..

[4] Gala FJ, Lupiani M, Raja R, Guillén C (2002). Actitudes psicológicas ante la muerte y el duelo. Una revisión conceptual. Cuadernos de Medicina Forense.

[5] Sansone RA, Sansone LA (2012). Physician grief with patient death. Innovations in clinical neuroscience.

[6] Soto-Perez-de-Celis E, Chavarri-Guerra Y, Pastrana T, Ruiz-Mendoza R, Bukowski A, Goss PE (2016). End-of-life Care in Latin America. J Glob Oncol.

[7] Pang SM, Sawada A, Konishi E (2003). A comparative study of Chinese, American and Japanese nurses’ perceptions of ethical role responsibilities. Nurs Ethics.

[8] Alfred D, Yarbrough S, Mink J (2013). Comparison of professional values of Taiwanese and United States nursing students. Nurs Ethics.

[9] Rassin MR (2010). Values grading among nursing students—differences between the ethnic groups. Nurs Educ Today.

[10] Bugen LA (1977). Human grief: a model for prediction and intervention. Am J Orthopsychiatry.

[11] Schmidt J (2007). Validación de la versión española de la escala de Bugen de afrontamiento de la muerte y de perfil revisado de actitudes hacia la muerte. Estudio comparativo y transcultural. Puesta en marcha de un programa de intervención. Tesis doctoral no publicada, Universidad de Granada.

[12] Robbins RA (1992). Death competency: a study of hospice volunteers. Death Studies.

[13] Bayés R, Limonero JT, Arranz P (2000). ¿Qué puede ayudarnos a morir en paz?. Rev Medicina Clínica.

[14] Centeno Cortés C, Núñez Olarte JM (1998). Estudios sobre la comunicación del diagnóstico de cáncer en España. Med Clin.

[15] Esteban de la Rosa MA. El enfermo terminal y la muerte: Problemas médico-sociales, propuestas de información médica y organización sanitaria. Tesis Doctoral. Universidad de Murcia. 1995.

[16] Rubio V, Sampedro E, Zapirain M, Gil I, Ayechu S, Tapiz V (2004). Diagnóstico: cáncer. ¿Queremos conocer la verdad?. Atención Primaria.

[17] Benbunan-Bentata B, Cruz-Quintana F, Roa-Venegas JM, Villaverde-Gutiérrez C (2007). Benbunan- Bentata BR. Afrontamiento del dolor y la muerte en estudiantes de Enfermería: una propuesta de intervención International Journal of Clinical and Health Psychology.

[18] Mercado R, García A (2016). Actitud hacia la muerte y su relación con la empatía médica en estudiantes de Medicina. Revista Cubana de Educación Médica Superior.

[19] Dueñas HJ, Corral J, Canchota E, Tovilla PM. Aspectos conductuales del médico frente al paciente moribundo y sus familiares. Rev Psiquiatra-s8a.com 2002. Vol 6, N° 3; Sep. Available at: http://www.xn--psiquiatra-s8a.com/psiquiatr%C3%ADa/revista/86/8817/?++interactivo [Accessed 20 May 2009].

[20] Cardozo R, Sosa M, Gómez A (2000). Apreciaciones sobre la Muerte en Estudiantes del Último Año de medicina.

[21] Limonero J (1997). Ansiedad ante la muerte. Univesidad Auntónoma de Barcelona. Facultad de psicología. Ansiedad y estrés.

[22] Motta I., Nuñes R., Cavalcanti T. Silva A., Gouveia V. Percepciones de estudiantes y médicos sobre la “muerte digna” Revista de bioét. 2016; 24 (1): 108–17.

[23] Gala León FJ, Lupiani JM, Raja HR, Guillén GC, González Infante JM (2002). Villaverde Gutiérrez Mª. C et al Actitudes psicológicas ante la muerte y el duelo: Una revisión conceptual Cuad med forense.

[24] Espinoza VM, Sanhueza A (2012). Miedo a la muerte y su relación con la inteligencia emocional DE estudiantes de enfermería de concepción. Acta Paul Enfermería.

[25] Grau J; Llantá M.; Massip C. Chacón M; Reyes M; Infante O; Romero T; Barroso I; Morales D. Ansiedad y actitudes ante la muerte: revisión y caracterización en un grupo heterogéneo de profesionales que se capacitan en cuidados paliativos. Instituto Nacional de Oncología y Radiobiología 2008. 4(1) 27–58.

[26] Huertas L, Allende S (2014). Creencias, actitudes y ansiedad ante la muerte en un equipo multidisciplinario de cuidados paliativos oncológicos. Psicooncología..

